# mTOR regulates T cell exhaustion and PD-1–targeted immunotherapy response during chronic viral infection

**DOI:** 10.1172/JCI160025

**Published:** 2023-01-17

**Authors:** Satomi Ando, Charles M. Perkins, Yamato Sajiki, Chase Chastain, Rajesh M. Valanparambil, Andreas Wieland, William H. Hudson, Masao Hashimoto, Suresh S. Ramalingam, Gordon J. Freeman, Rafi Ahmed, Koichi Araki

**Affiliations:** 1 Division of Infectious Diseases, Center for Inflammation and Tolerance, Cincinnati Children’s Hospital Medical Center, Cincinnati, Ohio, USA.; 2 Department of Pediatrics, University of Cincinnati College of Medicine, Cincinnati, Ohio, USA.; 3 Emory Vaccine Center,; 4 Depatment of Microbiology and Immunology, and; 5 Department of Hematology and Medical Oncology, Emory University School of Medicine, Atlanta, Georgia, USA.; 6 Winship Cancer Institute, Emory University, Atlanta, Georgia, USA.; 7 Department of Medical Oncology, Dana-Farber Cancer Institute, Department of Medicine, Harvard Medical School, Boston, Massachusetts, USA.

**Keywords:** Immunology, Infectious disease, Adaptive immunity, Immunotherapy, T cells

## Abstract

T cell exhaustion is a state of T cell dysfunction associated with expression of programmed death 1 (PD-1). Exhausted CD8^+^ T cells are maintained by self-renewing stem-like T cells that provide differentiated TIM3^+^ cells, a part of which possesses effector-like properties. PD-1–targeted therapies enhance T cell response by promoting differentiation of stem-like T cells toward TIM3^+^ cells, but the role of mTOR during T cell exhaustion remains elusive. Here, we showed that mTOR inhibition has distinct outcomes during the beginning of and after the establishment of chronic viral infection. Blocking mTOR during the T cell expansion phase enhanced the T cell response by causing accumulation of stem-like T cells, leading to improved efficacy of PD-1 immunotherapy; whereas, after exhaustion progressed, mTOR inhibition caused immunosuppression, characterized by decreased TIM3^+^ cells and increased viral load with minimal changes in stem-like T cells. Mechanistically, a cell-intrinsic mTOR signal was vital for differentiation of stem-like T cells into the TIM3^+^ state in the early and late phases of chronic infection as well as during PD-1 immunotherapy. Thus, PD-1 blockade worked after cessation of mTOR inhibition, but simultaneous treatment failed to induce functional TIM3^+^ cells, reducing efficacy of PD-1 immunotherapy. Our data demonstrate that mTOR regulates T cell exhaustion and have important implications for combination cancer therapies with PD-1 blockade.

## Introduction

T cell exhaustion is a state of T cell dysfunction; it is characterized by poor effector function, impaired proliferative capacity, and expression of multiple inhibitory receptors, most notably programmed death 1 (PD-1) (1, 2). Exhausted T cells can be found during chronic infection and in cancer, and blockade of the PD-1 pathway reinvigorates exhausted CD8^+^ T cells, resulting in better control of infection and cancer (1–5). Recent studies showed heterogeneity of exhausted CD8^+^ T cell populations and identified stem-like CD8^+^ T cells, characterized as PD-1^+^TIM3^–^TCF1^+^ (6–9). Stem-like CD8^+^ T cells not only undergo self-renewal but also provide more-differentiated TIM3^+^ cells that can be further divided into 2 subsets: effector-like transitory T cells and terminally exhausted T cells. These abilities of stem-like CD8^+^ T cells are essential to maintain antigen-specific CD8^+^ T cells during chronic infection. Despite such memory T cell–like properties, stem-like CD8^+^ T cells are transcriptionally and epigenetically distinct from memory CD8^+^ T cells that arise during acute infection and appear to be generated as a result of adaptation to chronic antigen stimulation (10–12). Stem-like CD8^+^ T cells are present not only during chronic infection but also in human cancers (13–18), and PD-1 blockade increases the number of effector-like transitory T cells by promoting the differentiation of stem-like T cells toward the TIM3^+^ state (6, 19, 20). In contrast, terminally exhausted CD8^+^ T cells minimally divide after PD-1–targeted immunotherapy. This heterogeneity of exhausted CD8^+^ T cell populations emphasizes the need for a better understanding of the generation and maintenance of these subsets to optimize treatment strategies when these cells are harnessed.

mTOR is a serine/threonine kinase that regulates several key cellular processes, including translation, autophagy, and metabolism (21, 22). Rapamycin, a specific inhibitor of mTOR, and its analogs are currently used as immunosuppressive drugs in transplant recipients (23, 24). Rapamycin-mediated immunosuppression is generally thought to diminish proliferation of alloantigen-specific T cells, but recent studies suggest that it efficiently inhibits humoral immunity, leading to impaired antibody responses (25–30). In contrast to such an immunosuppressive effect, rapamycin can stimulate memory CD8^+^ T cell formation during acute infection and vaccination when antigen is cleared (31, 32). However, although several studies examined CD8^+^ T cell responses in the presence of rapamycin during chronic infection (33, 34), it remains unclear how inhibition of mTOR in antigen-specific CD8^+^ T cells modulates differentiation of stem-like T cells into TIM3^+^ cells as well as how mTOR inhibition affects PD-1 blockade therapy during CD8^+^ T cell exhaustion. Because mTOR inhibitors and drugs targeting molecules upstream of mTOR, such as PI3K inhibitors, are approved for treatment of cancer (35–37), it is essential to elucidate the role of mTOR in T cell exhaustion to further improve immunotherapy for patients with cancer.

To address this issue, we investigated the effect of rapamycin on virus-specific CD8^+^ T cells in a mouse model of chronic viral infection with lymphocytic choriomeningitis virus (LCMV). We found that drug treatment during the T cell expansion phase of chronic viral infection enhanced the number of virus-specific CD8^+^ T cells, an effect similar to that of rapamycin during acute viral infection. Notably, inhibition of mTOR promoted the formation of stem-like CD8^+^ T cells, leading to improved efficacy of subsequent PD-1–targeted immunotherapy. On the other hand, when T cell exhaustion was fully established, the drug suppressed CD8^+^ T cell immunity by decreasing the number of more-differentiated TIM3^+^CD8^+^ T cells. To further dissect the mechanism of mTOR-mediated CD8^+^ T cell responses during chronic infection, RNA-Seq analyses and shRNA experiments were conducted. Overall, we showed that T cell–intrinsic mTOR is required for differentiation of stem-like T cells into a TIM3^+^ state in both the early and late stages of chronic infection and is essential for PD-1 blockade–mediated induction of functional TIM3^+^CD8^+^ T cells. Our work has important implications for combination cancer therapies with PD-1.

## Results

### Rapamycin enhances CD8^+^ T cell response by promoting the formation of stem-like T cells during the early phase of chronic infection.

**Figure 1:**
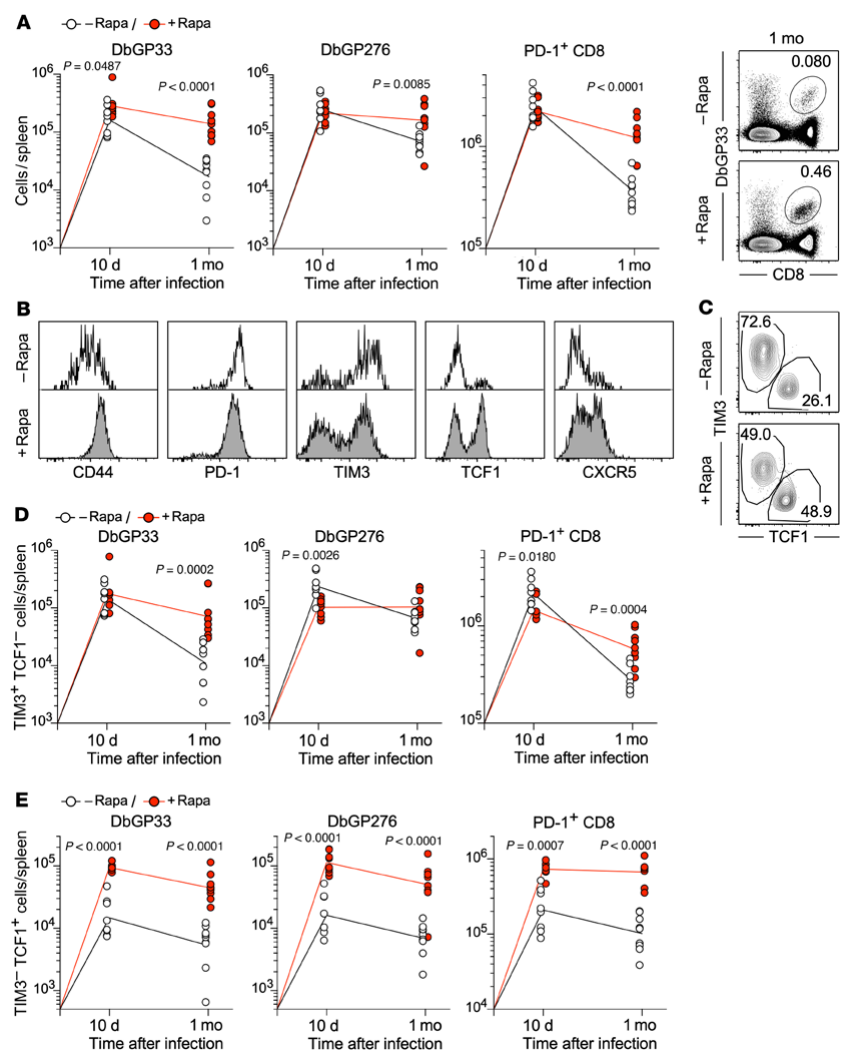
mTOR inhibition enhances CD8^+^ T cell response by promoting the formation of stem-like CD8^+^ T cells during the early phase of chronic infection. Mice were infected with LCMV clone 13 in the presence or absence of rapamycin treatment. Rapamycin was injected i.p. every day from day –1 to day 30–35 (1 month) after infection. LCMV-specific CD8^+^ T cell response was examined on days 10 and 31–36 (1 month) after infection. (**A**) The total number of DbGP33 tetramer^+^, DbGP276 tetramer^+^, and PD-1^+^CD8^+^ T cells in the spleen at 10 days and 1 month after infection (*n* = 8 per each group for 10 days, *n* = 9 per each group for 1 month). Flow cytometry plots, gated on total live splenocytes, show the frequency of DbGP33 tetramer^+^ CD8^+^ T cells 1 month after infection. (**B**) Phenotypic analysis of LCMV-specific DbGP33 tetramer^+^ CD8^+^ T cells in the spleen at 1 month after infection. (**C**) The frequency of stem-like (TIM3^–^TCF1^+^) and TIM3^+^ more-differentiated (TIM3^+^TCF1^–^) LCMV-specific CD8^+^ T cells in the spleen at 1 month after infection. The flow cytometry plots were gated on DbGP33 tetramer^+^ CD8^+^ T cells. (**D**) The number of TIM3^+^ differentiated (TIM3^+^TCF1^–^) or (**E**) stem-like (TIM3^–^TCF1^+^) CD8^+^ T cells in the spleen is shown for DbGP33 tetramer^+^, DbGP276 tetramer^+^, and PD-1^+^CD8^+^ T cells (*n* = 8 per each group for 10 days, *n* = 9 per each group for 1 month). Each symbol represents an individual mouse. Each line represents geometric means, and *P* values were calculated by unpaired *t* test. Data were pooled from 2 or 3 independent experiments.

To understand how the mTOR pathway is involved in the exhausted CD8^+^ T cell response, mice were treated with rapamycin daily during the entire course of chronic LCMV infection, and virus-specific CD8^+^ T cells were examined 10 days and 1 month after infection (Figure 1). We observed a comparable number of DbGP33 and DbGP276 tetramer^+^ CD8^+^ T cells as well as PD-1^+^CD8^+^ T cells, most of which are LCMV specific, 10 days after infection between control and rapamycin-treated mice (Figure 1A). However, rapamycin treatment increased the number of LCMV-specific CD8^+^ T cells 1 month after infection (Figure 1A). This increase was not due to dampened migration of these cells to peripheral tissues, because equal or slightly higher numbers of them were detected in livers and lungs in rapamycin-treated mice (Supplemental Figure 1; supplemental material available online with this article; https://doi.org/10.1172/JCI160025DS1). We next compared the phenotype of LCMV-specific CD8^+^ T cells between the rapamycin-treated and untreated mice. CD44, TCF1, and CXCR5 were upregulated by rapamycin treatment, and, conversely, TIM3 was downregulated (Figure 1B). These phenotypic differences strongly suggest that rapamycin modulates the formation of 2 distinct subsets that arise during chronic infection: stem-like CD8^+^ T cells (PD-1^+^TIM3^–^TCF1^+^) and more differentiated TIM3^+^CD8^+^ T cells (PD-1^+^TIM3^+^TCF1^–^). To test this, antigen-specific CD8^+^ T cells were stained for the markers TIM3 and TCF1. We found that antigen-specific CD8^+^ T cells in the presence of rapamycin showed an increased frequency of stem-like (TIM3^–^TCF1^+^) CD8^+^ T cells compared with those from control mice (Figure 1C). At 10 days after infection, we observed significant augmentation of stem-like CD8^+^ T cell numbers and a slight reduction of the more-differentiated TIM3^+^TCF1^–^ population in rapamycin-treated mice (Figure 1, D and E), accompanied by increased viral load (Supplemental Figure 2). At 1 month after infection, the quantity of both subsets was higher in the rapamycin-treated group compared with the control group (Figure 1, D and E), but, most notably, stem-like CD8^+^ T cell numbers in rapamycin-treated mice were strikingly higher than those in untreated mice (Figure 1E). There was no difference in viral titer 1 month after infection between rapamycin-treated and untreated mice (Supplemental Figure 2). Furthermore, the lower dose of rapamycin, which was used in previous studies during acute infection (29, 31, 38), also enhanced antigen-specific CD8^+^ T cell responses to a comparable extent as the higher dose of rapamycin used in Figure 1 (Supplemental Figure 3). Taken together, these results indicate that inhibition of the mTOR pathway with rapamycin during the T cell expansion phase promoted the generation of stem-like CD8^+^ T cells, leading to the higher quantity of antigen-specific CD8^+^ T cells in rapamycin-treated mice compared with control mice.

### Transcriptional signatures reveal characteristics of stem-like and more-differentiated TIM3^+^CD8^+^ T cells generated in rapamycin-treated mice.

**Figure 2:**
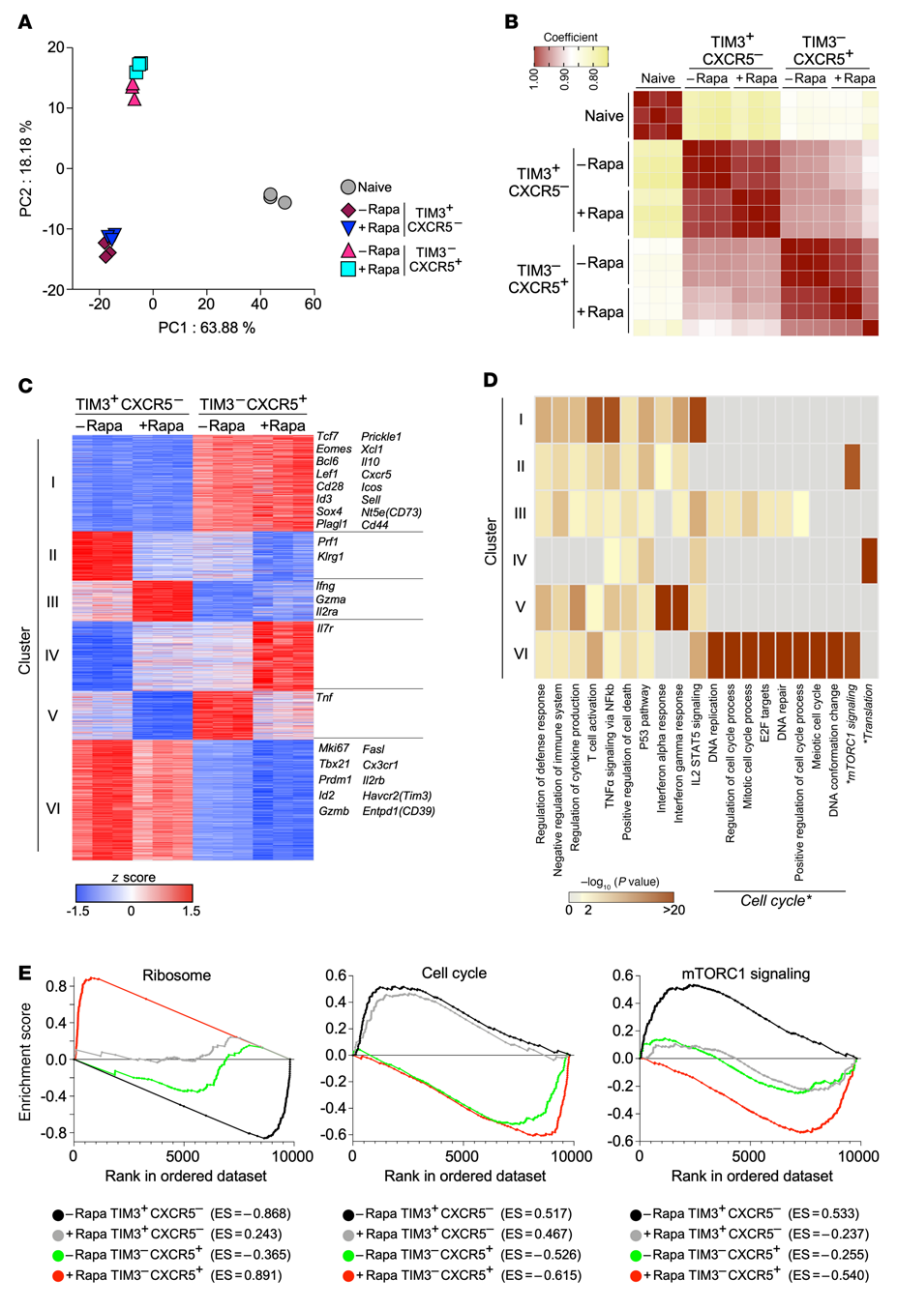
Transcriptional signatures of stem-like and TIM3^+^ differentiated CD8^+^ T cells generated in rapamycin-treated mice. LCMV clone 13–infected mice were treated or not with rapamycin from day –1 to day 9. At day 10 after infection, stem-like (TIM3^–^CXCR5^+^PD-1^+^) or TIM3^+^ more-differentiated (TIM3^+^CXCR5^–^PD-1^+^) CD8^+^ T cells were sorted, and RNA-Seq was performed. Naive CD8^+^ T cells (CD44^lo^CD8^+^) from uninfected mice were sorted for RNA-Seq as a control. See Supplemental Figure 4 for gating strategy. (**A**) Principal component analysis. (**B**) Heatmap illustrating Spearman’s correlation among individual samples. (**C**) Heatmap showing the relative expression (*z* score) of the top 3,000 genes that were differentially expressed among 4 populations (TIM3^+^ more-differentiated (TIM3^+^CXCR5^–^) CD8^+^ T cells [rapamycin vs. control] and stem-like (TIM3^–^CXCR5^+^) CD8^+^ T cells [rapamycin vs. control]). Genes were divided into 6 clusters by K-means clustering based on expression. (**D**) Metascape analysis showing the biological processes associated with genes in the 6 clusters of genes shown in **C**. The biological processes marked by asterisks were described in the main text and further analyzed in **E**. (**E**) Gene set enrichment analysis (GSEA) of TIM3^+^ differentiated (TIM3^+^CXCR5^–^) and stem-like (TIM3^–^CXCR5^+^) CD8^+^ T cells from rapamycin-treated and untreated mice using gene sets for ribosome, cell cycle, and mTORC1 signaling. GSEA was performed by comparing gene expression data from each cell population with the combined data from the remaining 3 other populations. Data are from 3 independent experiments with samples pooled from 4 to 6 mice for sorting individual cell populations.

Our data showed that rapamycin treatment during the T cell expansion phase enhanced the formation of stem-like CD8^+^ T cells (Figure 1). Next, to examine whether transcriptional signatures of the 2 distinct CD8^+^ T cell subsets are altered by mTOR inhibition, we performed RNA-Seq analyses of stem-like and TIM3^+^ differentiated CD8^+^ T cells that were sorted from LCMV-infected mice in the presence or absence of rapamycin treatment (Supplemental Figure 4). To compare the overall transcriptional profiles of these antigen-specific CD8^+^ T cell populations (Supplemental Table 1, normalized gene counts), principal component analysis (PCA) was carried out (Figure 2A). The gene expression signatures of stem-like CD8^+^ T cells generated in rapamycin-treated mice were similar to those in control mice. Likewise, more-differentiated TIM3^+^CD8^+^ T cells from the rapamycin-treated mice possessed gene expression profiles similar to those of controls. In accordance with the PCA analysis, Spearman’s correlation showed high coefficient factors of more than 0.95 in each subset between rapamycin-treated and untreated mice (compare stem-like CD8^+^ T cells [TIM3^–^CXCR5^+^] between rapamycin treated and untreated and compare more-differentiated CD8^+^ T cells [TIM3^+^CXCR5^–^] between rapamycin treated and untreated) (Figure 2B). These transcriptional profiles demonstrate that the landscape of global gene expression signatures of antigen-specific CD8^+^ T cells generated during rapamycin treatment resembles that of control mice.

Next, to further examine expression of individual genes in LCMV-specific CD8^+^ T cells, we selected the top 3,000 genes (Supplemental Table 2) that were differentially expressed among the 4 populations (stem-like CD8^+^ T cells [rapamycin treated vs. control] and differentiated TIM3^+^CD8^+^ T cells [rapamycin treated vs. control] and divided them into 6 clusters by K-means clustering (Figure 2C). Genes in clusters I and VI were differentially expressed between stem-like and TIM3^+^ differentiated CD8^+^ T cells but not between rapamycin-treated and control groups. These clusters contained canonical markers, which were identified in a previous study (6), for stem-like and TIM3^+^ differentiated CD8^+^ T cells, supporting the data obtained in the PCA and Spearman’s correlation analyses (Figure 2, A and B). However, other clusters (II, III, IV, V) showed different expression patterns in each subset between rapamycin-treated and control groups. In differentiated TIM3^+^CD8^+^ T cells, *Prf1* and *Klrg1* were downregulated by rapamycin treatment, whereas *Ifng*, *Gzma*, and *IL2ra* were upregulated (Figure 2C). In stem-like CD8^+^ T cells, rapamycin treatment enhanced *IL7r* expression and conversely decreased *Tnf* (Figure 2C). Next, to investigate what biological activity and/or pathways are enriched in the genes of individual clusters, we analyzed these genes by Metascape (39) to determine gene ontology categories overrepresented in the sets of genes (Figure 2D). Notably, genes in cluster II were enriched by mTORC1 signaling. Because genes in this cluster were downregulated in the presence of rapamycin in each subset (Figure 2, C and D), these data indicate that rapamycin treatment inhibited expression of mTORC1 signaling–associated genes. In contrast, substantial enrichment for genes related to translation, those mostly encoding ribosomal proteins, was observed in cluster IV (Figure 2D and Supplemental Table 2), suggesting that ribosomal protein mRNAs are highly expressed in stem-like CD8^+^ T cells from rapamycin-treated mice (see cluster IV in Figure 2C). We also found that multiple gene sets related to cell cycle were significantly enriched in cluster VI (Figure 2D); genes from these subsets were upregulated in TIM3^+^ more-differentiated CD8^+^ T cells (see cluster VI in Figure 2C).

We further investigated the gene expression involved in biological themes/pathways identified in Figure 2D (translation/ribosomes, cell cycle, and mTORC1 signaling) using gene set enrichment analysis (GSEA). First, we observed striking enrichment of a gene set associated with ribosomes in stem-like CD8^+^ T cells generated in rapamycin-treated mice (Figure 2E, ribosome, red). In contrast, more-differentiated TIM3^+^CD8^+^ T cells in the control group showed substantial downregulation of these genes (Figure 2E, ribosome, black). Interestingly, TIM3^+^ differentiated CD8^+^ T cells obtained from the rapamycin group had a higher enrichment score compared with that of stem-like CD8^+^ T cells in the absence of rapamycin (Figure 2E, ribosome, compare gray and green). Second, cell-cycle–related genes were examined, and we found that TIM3^+^ more-differentiated CD8^+^ T cells in both rapamycin-treated and control groups highly expressed cell-cycle–related genes compared with stem-like CD8^+^ T cells (Figure 2E, cell cycle). Enrichment scores for this gene set were slightly lower in the cells derived from the rapamycin-treated groups (Figure 2E, cell cycle, compare black and gray, also compare green and red). Finally, the mTORC1 signaling pathway was analyzed by GSEA. TIM3^+^ differentiated CD8^+^ T cells in control mice showed substantial enrichment in mTORC1 signaling–related genes relative to those in rapamycin-treated mice (Figure 2E, mTORC1 signaling, compare black and gray). In contrast, these genes were considerably downregulated in stem-like CD8^+^ T cells in rapamycin-treated mice compared with those in the other 3 groups (Figure 2E, mTORC1 signaling, red). Taken together, these results show that inhibition of mTOR with rapamycin alters several biological themes/pathways in antigen-specific CD8^+^ T cells. Specifically, downregulation of genes related to mTORC1 signaling in antigen-specific CD8^+^ T cells derived from the rapamycin-treated group suggests that the drug inhibits the mTOR pathway intrinsically in antigen-specific CD8^+^ T cells.

### mTOR acts intrinsically in antigen-specific CD8^+^ T cells to regulate differentiation of stem-like T cells into a TIM3^+^ more-differentiated state during T cell exhaustion.

**Figure 3:**
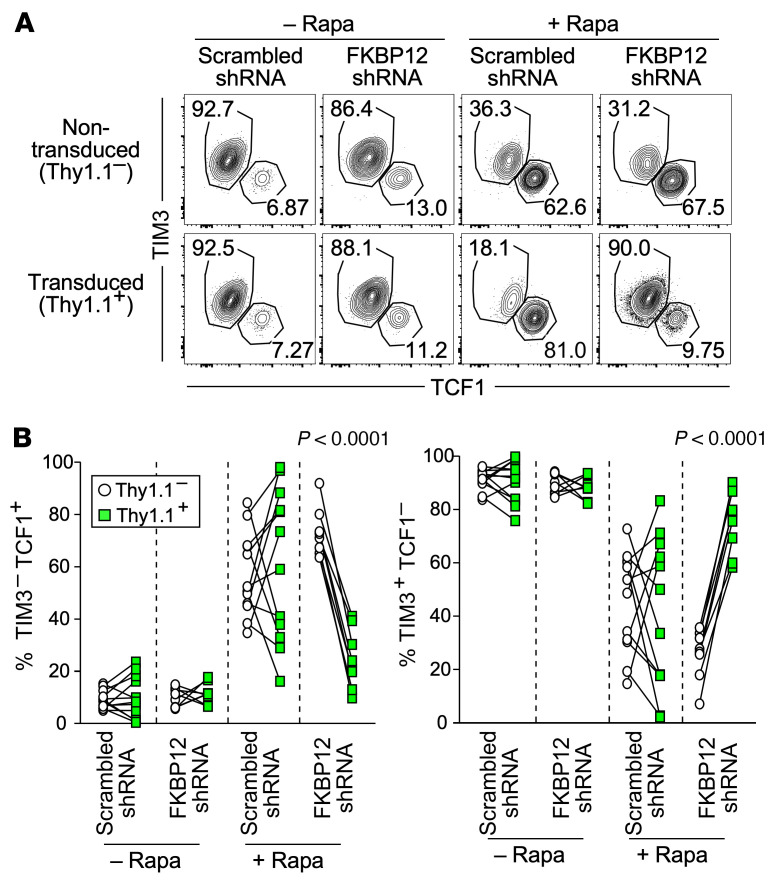
mTOR acts intrinsically in antigen-specific CD8^+^ T cells to regulate T cell exhaustion during chronic infection. LCMV-specific transgenic CD8^+^ T cells (P14 cells) were transduced with retrovirus expressing FKBP12 shRNA or scrambled shRNA. These retrovirus-transduced P14 cells (marked by Thy1.1) and nontransduced P14 cells were adoptively cotransferred into rapamycin-treated or untreated B6 mice followed by LCMV clone 13 infection. Rapamycin was i.p. administered every day from day –1 to day 9 after infection. See experimental design in Supplemental Figure 5. P14 cells were analyzed at day 10 after infection. (**A**) The flow cytometry plots, gated on retrovirus-transduced (Thy1.1^+^) or nontransduced (Thy1.1^–^) cells, show the frequency of stem-like (TIM3^–^TCF1^+^) and TIM3^+^ differentiated (TIM3^+^TCF1^–^) retrovirus-transduced and nontransduced P14 T cells in the spleen. (**B**) The frequency of stem-like (TIM3^–^TCF1^+^) and TIM3^+^ differentiated (TIM3^+^TCF1^–^) CD8^+^ T cells in retrovirus- transduced (green squares) and nontransduced (white circles) P14 cells in the spleen. *n* = 12 per each group for scrambled shRNA in the presence or absence of rapamycin. *n* = 8 per each group for FKBP12 shRNA in the presence or absence of rapamycin. Each line represents a comparison between nontransduced and transduced P14 cells in the same mice. Data were pooled from 4 independent experiments. *P* values were calculated by paired *t* test.

Our rapamycin treatment data clearly show that mTOR plays a crucial role in T cell responses during chronic viral infection. The RNA-Seq analyses imply that rapamycin may suppress mTOR activity intrinsically in antigen-specific CD8^+^ T cells to regulate T cell exhaustion. To address this, we knocked down FKBP12 in antigen-specific CD8^+^ T cells using a retrovirus-based shRNA system. FKBP12 is an essential intracellular binding partner of rapamycin, and the FKBP12-rapamycin complex inhibits the mTORC1 pathway (40–42). Thus, by knocking down FKBP12 in antigen-specific CD8^+^ T cells, we rendered these cells insensitive to rapamycin-mediated mTOR inhibition. Scrambled shRNA– or FKBP12 shRNA–expressing retrovirus-transduced LCMV-specific transgenic CD8^+^ T cells identified by the Thy-1.1 marker and nontransduced transgenic CD8^+^ T cells were adoptively cotransferred into recipient mice followed by LCMV infection in the presence or absence of rapamycin (Supplemental Figure 5). This system allowed us to examine intrinsic effects of rapamycin by comparing Thy-1.1^+^ transduced cells with Thy-1.1^–^ nontransduced cells in the same mouse. As shown in Figure 3, FKBP12 knockdown itself, without rapamycin treatment, did not change the phenotype of antigen-specific CD8^+^ T cells 10 days after infection. However, the effect of rapamycin treatment on antigen-specific CD8^+^ T cells was lost by FKBP12 knockdown. Thus, in rapamycin-treated mice, the stem-like CD8^+^ T cell population significantly decreased after knockdown of FKBP12 in antigen-specific CD8^+^ T cells compared with that in nontransduced or scrambled shRNA–transduced cells, whereas the TIM3^+^ more-differentiated CD8^+^ T cell population increased after FKBP12 knockdown (Figure 3). Furthermore, these phenotypic changes in FKBP12-knockdown antigen-specific CD8^+^ T cells from rapamycin-treated mice were also observed 1 month after infection (Supplemental Figure 6). These data demonstrate that rapamycin acted intrinsically in antigen-specific CD8^+^ T cells to regulate the formation of the 2 subsets.

### T cells induced by rapamycin treatment at the beginning of chronic infection improves efficacy of PD-1–targeted therapy.

**Figure 4:**
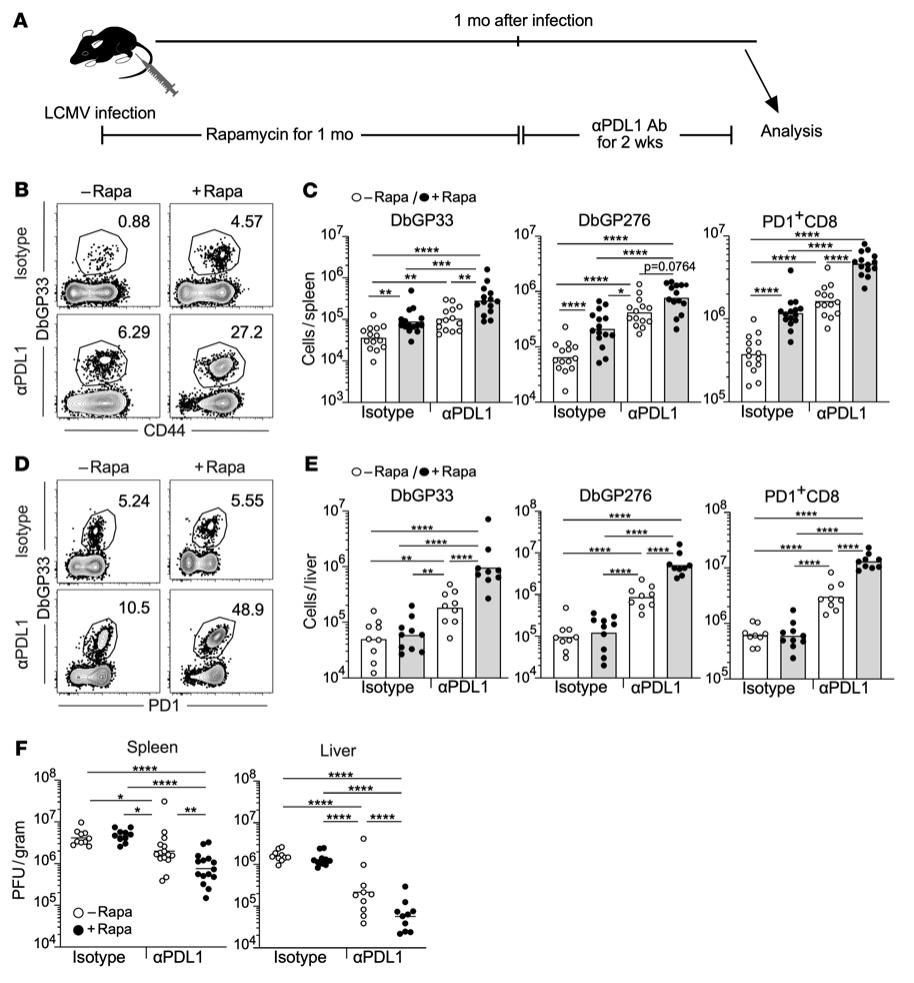
Rapamycin treatment during the beginning of chronic infection improves efficacy of PD-1–targeted therapy. (**A**) Experimental design. Mice were infected with LCMV clone 13 in the presence or absence of rapamycin treatment. Rapamycin was i.p. administered every day from day –1 to day 33–36 of infection. Treatment with anti-PDL1 antibody or isotype control antibody was started from the day after rapamycin discontinuation, and these antibodies were i.p. injected every 3 days, for a total of 5 injections. Immune response and viral titer were analyzed at day 14 after PD-1 blockade was started. (**B** and **D**) The frequency of DbGP33 tetramer^+^ CD8^+^ T cells. Flow cytometry plots were gated on CD8^+^ T cells. (**C** and **E**) The number of DbGP33 tetramer^+^, DbGP276 tetramer^+^, and PD-1^+^CD8^+^ T cells (*n* = 14 per each group except for the isotype antibody group treated with rapamycin [*n* = 15] for spleen, *n* = 9 per each group except for the isotype antibody group treated with rapamycin [*n* = 10] for liver). Spleen data are shown in **B** and **C**, and liver data are shown in **D** and **E**. (**F**) Viral titers in the spleen and liver. Spleen: isotype with and without rapamycin (*n* = 10 per each group), anti–PD-L1 with and without rapamycin (*n* = 15 per each group). Liver: *n* = 10 per each group. Each symbol represents an individual mouse. Each bar in **C** and **E** and each horizontal line in **F** represent geometric means. *P < 0.05; **P < 0.01; ***P < 0.001; ****P < 0.0001 by 1-way ANOVA. Data were pooled from 2 or 3 independent experiments.

Next, we asked whether the increased number of stem-like CD8^+^ T cells induced by rapamycin has a beneficial effect on PD-1–targeted therapy because this cell population responds to and substantially proliferates after blocking PD-1 signaling (6). To test this, we administered anti–PD-L1 antibody to LCMV-infected mice after ceasing rapamycin treatment (Figure 4A). PD-1 blockade increased the quantity of antigen-specific CD8^+^ T cells (DbGP33 tetramer^+^, DbGP276 tetramer^+^, and PD-1^+^ cells) compared with isotype controls in both spleens and livers (Figure 4, B–E). The number of expanded antigen-specific CD8^+^ T cells caused by PD-1 blockade was significantly higher in rapamycin-treated mice relative to that in mice that did not receive rapamycin (compare rapamycin-treated and untreated mice in the anti–PD-L1 antibody groups, Figure 4, B–E). In accordance with the CD8^+^ T cell response results, PD-1 blockade resulted in a significant reduction in viral loads in spleens and livers of rapamycin-treated mice compared with control mice (Figure 4F). Next, to examine if rapamycin treatment improves the quality of stem-like CD8^+^ T cells, we compared the responsiveness of stem-like CD8^+^ T cells generated in rapamycin-treated or untreated mice to PD-1 blockade. CD45.2^+^ stem-like CD8^+^ T cells generated in the presence or absence of rapamycin were adoptively transferred into chronically infected recipient mice (CD45.1^+^), followed by treatment with anti–PD-L1 antibody (Supplemental Figure 7A). As shown in Supplemental Figure 7B, both rapamycin-exposed and rapamycin-naive transferred stem-like CD8^+^ T cells similarly responded to anti–PD-L1 antibody treatment, suggesting that mTOR inhibition does not alter the quality of stem-like CD8^+^ T cells in their responsiveness to PD-1 blockade. Taken together, these results show that mTOR inhibition enhances the quantity of stem-like CD8^+^ T cells, leading to improved efficacy of PD-1–targeted therapy.

### mTOR inhibition impairs antiviral T cell immunity by decreasing the generation of effector-like transitory T cells from stem-like T cells after fully establishing T cell exhaustion.

**Figure 5:**
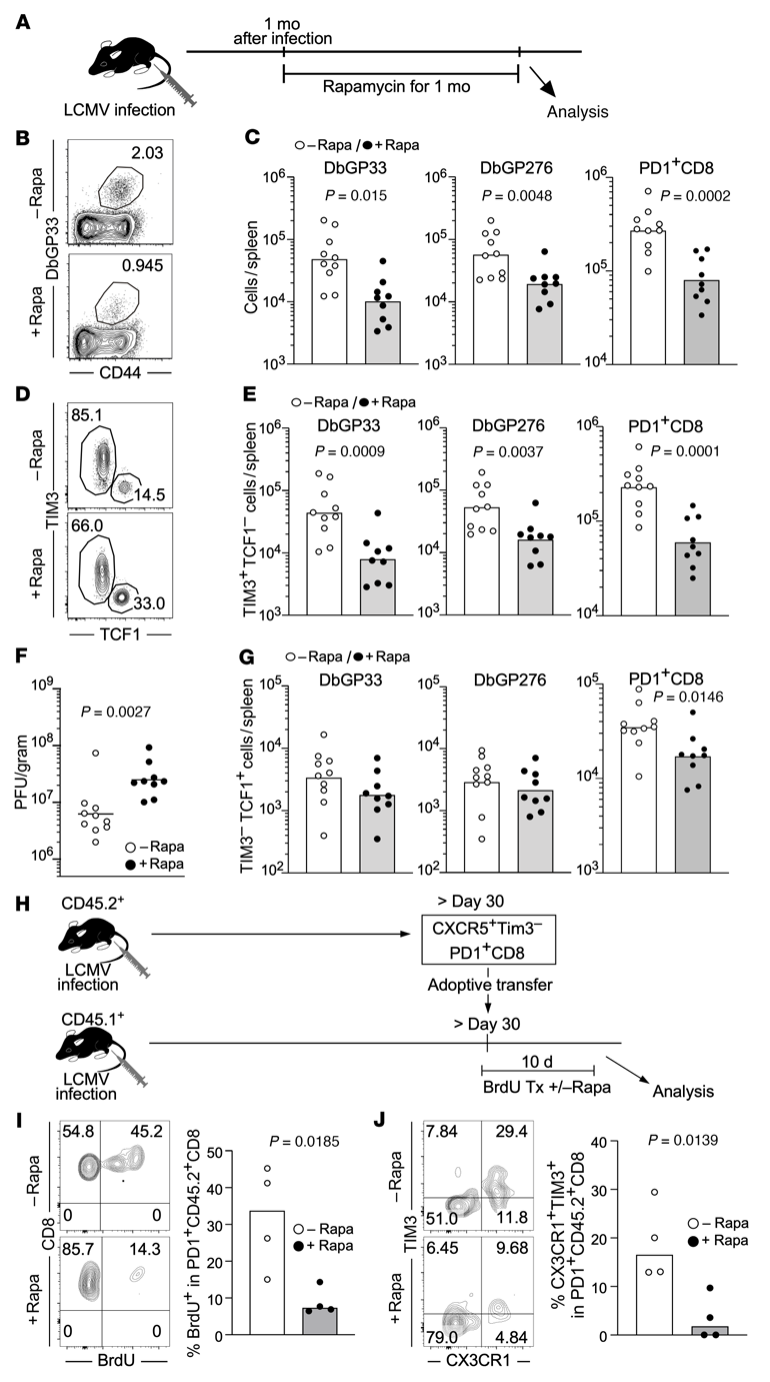
mTOR inhibition impairs antiviral T cell immunity by decreasing the generation of effector-like transitory T cells from stem-like T cells after establishing chronic infection. (**A**–**G**) Mice were infected with LCMV clone 13, and then rapamycin was administered daily i.p. to the mice from day 31 or 36 to day 62 or 67, respectively. Analyses were performed in spleens 1 month after rapamycin treatment. (**A**) Experimental design. (**B**) The frequency of DbGP33^+^ cells in the spleen (gated on CD8). (**C**) The total number of DbGP33^+^, DbGP276^+^, and PD-1^+^CD8^+^ T cells in the spleen. (**D**) The frequency of stem-like (TIM3^–^TCF1^+^) and TIM3^+^ differentiated (TIM3^+^TCF1^–^) cells in the spleen (gated on DbGP33^+^ CD8). (**E**) The number of TIM3^+^ differentiated (TIM3^+^TCF1^–^) or (**G**) stem-like (TIM3^–^TCF1^+^) CD8^+^ T cells in the spleen is shown for DbGP33^+^, DbGP276^+^, and PD-1^+^CD8^+^ T cells. (**F**) Viral titers in spleens. (**C** and **E**–**G**) *n* = 10 for control and *n* = 9 for rapamycin group. (**H**–**J**) Stem-like CD8^+^ T cells (TIM3^–^CXCR5^+^PD1^+^) were sorted from spleens of chronically infected mice (CD45.2^+^) at more than 30 days after infection. Sorted cells were transferred into chronically infected mice (>day 30 after infection, CD45.1^+^). BrdU-containing drinking water was given recipient mice. Rapamycin was administered daily for 10 days starting 1 day prior to adoptive transfer. (**H**) Experimental design. Tx, treatment. (**I** and **J**) The frequency of (**I**) BrdU^+^ cells and (**J**) effector-like transitory (CX3CR1^+^TIM3^+^) cells in PD1^+^CD45.2^+^CD8^+^ T cells in the spleen (flow plots, gated on PD1^+^CD45.2^+^ CD8). Each symbol represents an individual mouse. Each bar in **C** and **E**–**G** and each horizontal line in **F** represents geometric means. Each bar in **I** and **J** represents means. *P* values were calculated by unpaired *t* test. Data were pooled from 2 independent experiments.

Next, in order to examine the role of mTOR in antigen-specific CD8^+^ T cells after fully establishing T cell exhaustion, rapamycin was administered to LCMV chronically infected mice for 30 days from 1 month to 2 months after infection (Figure 5A). In contrast to that after treatment in the early phase of infection, we observed significantly lower numbers of virus-specific CD8^+^ T cells in rapamycin-treated mice (Figure 5, B and C). This reduction of total cell number was due to a substantial decrease in TIM3^+^ differentiated CD8^+^ T cells (Figure 5, D and E). Furthermore, the quantity of antigen-specific CD8^+^ T cells negatively correlated with viral loads, and we observed higher viral titers in rapamycin-treated mice compared with untreated mice (Figure 5F). Despite such negative effects of rapamycin on TIM3^+^ differentiated CD8^+^ T cells and viral loads, the drug had minimal or no effect on the number of stem-like CD8^+^ T cells compared with controls (Figure 5G). These data suggest that rapamycin may prevent stem-like CD8^+^ T cells from differentiating into CX3CR1^+^TIM3^+^ effector-like transitory CD8^+^ T cells, which are important for viral control during chronic infection (19, 20). To examine this, CD45.2^+^ stem-like CD8^+^ T cells were isolated from mice chronically infected with LCMV, and these cells were adoptively transferred into chronically infected recipient mice (CD45.1^+^) in the presence or absence of rapamycin treatment (Figure 5H). Because differentiation into effector-like transitory CD8^+^ T cells is accompanied by proliferation, we gave the mice BrdU to monitor cell proliferation (Figure 5H). Rapamycin treatment decreased proliferation of the transferred cells, as shown by lower BrdU incorporation compared with that in control mice (Figure 5I). The generation of CX3CR1^+^TIM3^+^ effector-like transitory CD8^+^ T cells from the transferred stem-like T cells was significantly inhibited by rapamycin treatment (Figure 5J). Collectively, our results show that mTOR inhibition after fully establishing T cell exhaustion impairs T cell immunity by inhibiting generation of effector-like transitory CD8^+^ T cells from stem-like T cells.

### Proliferative capacity and susceptibility to mTOR inhibition differ between naive and chronically stimulated CD8^+^ T cells.

Our data showed that rapamycin treatment inhibited the differentiation of antigen-specific CD8^+^ T cells into the TIM3^+^ more-differentiated state during the beginning of chronic infection as well as after establishment of T cell exhaustion. However, mTOR inhibition had distinct outcomes in terms of the number of antigen-specific CD8^+^ T cells between these early and late time points of infection. When rapamycin was administered from the time of infection, the number of antigen-specific CD8^+^ T cells increased (Figure 1). On the other hand, rapamycin treatment after establishment of chronic infection decreased their quantity (Figure 5). Such discrepancies in antigen-specific CD8^+^ T cell numbers with rapamycin treatment may occur due to qualitative differences between naive and chronically stimulated CD8^+^ T cells. We therefore examined the effect of rapamycin on proliferative potential of naive and chronically stimulated antigen-specific CD8^+^ T cells. Splenic single-cell suspensions containing naive or chronically stimulated LCMV GP33 epitope-specific TCR transgenic CD8^+^ T cells (P14 cells) were cultured with GP33 peptide and IL-2 for 1 week (Supplemental Figure 8A). Both P14 cell populations expanded during the 1-week stimulation period, but the frequency of P14 cells derived from chronically infected mice was significantly lower than that of naive P14 cells (Supplemental Figure 8B). Furthermore, we found that the chronically stimulated P14 cell population was highly sensitive to mTOR inhibition. Thus, while rapamycin had no negative effect on the number of naive P14 cells, the drug dose-dependently inhibited expansion of P14 cells derived from chronically infected mice (Supplemental Figure 8C). These data clearly show that the proliferative capacity and susceptibility to mTOR inhibition differ between naive and chronically stimulated CD8^+^ T cells. This might contribute to the different effects on the number of antigen-specific CD8^+^ T cells in vivo when rapamycin was administered during the T cell priming phase versus after establishment of chronic infection.

### mTOR is required for the generation of effector-like transitory T cells induced by PD-1–targeted immunotherapy.

**Figure 6:**
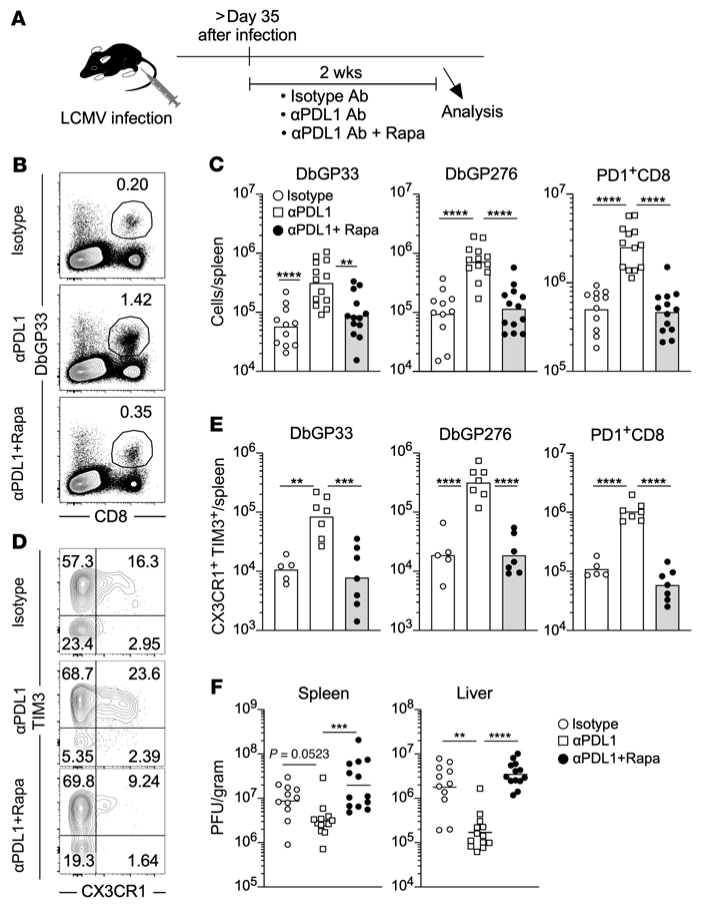
mTOR is required for the generation of effector-like transitory T cells induced by PD-1–targeted immunotherapy. (**A**) Experimental design. Mice were infected with LCMV clone 13, and then rapamycin treatment and PD-1 blockade was started simultaneously after establishing chronic infection. Rapamycin was administered daily i.p. to LCMV chronically infected mice and anti–PD-L1 antibody (αPDL1) or isotype control antibody (Isotype) was i.p. administered every 3 days, for a total of 5 injections. Immune response and viral titer were analyzed 2 days after final injection of antibodies. (**B**) The flow cytometry plots, gated on total live splenocytes, show the frequency of DbGP33 tetramer^+^ CD8^+^ T cells in the spleen. (**C**) The number of DbGP33 tetramer^+^, DbGP276 tetramer^+^, and PD-1^+^CD8^+^ T cells in spleens. *n* = 11 for isotype control, *n* = 13 for αPDL1 alone and aPDL1 plus rapamycin group. (**D**) The flow cytometry plots, gated on DbGP33 tetramer^+^ CD8^+^ T cells in the spleen, show the frequency of effector-like transitionary (CX3CR1^+^TIM3^+^) CD8^+^ T cells. (**E**) The number of effector-like transitionary (CX3CR1^+^TIM3^+^) DbGP33 tetramer^+^, DbGP276 tetramer^+^, and PD-1^+^ CD8^+^ T cells in spleens. *n* = 5 for isotype control, *n* = 7 for αPDL1 alone and αPDL1 plus rapamycin group. (**F**) Viral titer in the spleen and liver. *n* = 11 for isotype control, *n* = 13 for αPDL1 alone and αPDL1 plus rapamycin group in the spleen and liver. Each symbol represents an individual mouse. Each bar in **C** and **E** and each horizontal line in **F** represent geometric means. **P < 0.01; ***P < 0.001; ****P < 0.0001 (1-way ANOVA). Data were pooled from 2 or 3 independent experiments.

The data in Figure 5 show that mTOR was vital for generation of effector-like transitory CD8^+^ T cells from stem-like T cells after fully establishing T cell exhaustion. PD-1 blockade increases the number of effector-like transitory T cells by promoting this differentiation process (19, 20). Thus, we next examined if mTOR is required for PD-1 blockade–mediated induction of effector-like transitory CD8^+^ T cells during chronic viral infection (Figure 6). LCMV-infected mice were simultaneously treated with anti–PD-L1 antibody and rapamycin, and the obtained data were compared with those from isotype- and anti–PD-L1 antibody–treated LCMV-infected mice (Figure 6A). The number of antigen-specific CD8^+^ T cells derived from the rapamycin plus anti–PD-L1 antibody–treated mice significantly decreased relative to that from the mice receiving anti–PD-L1 antibody alone and was comparable to that from isotype control mice (Figure 6, B and C). Furthermore, simultaneous treatment blocked the generation of CX3CR1^+^TIM3^+^ effector-like transitory CD8^+^ T cells (Figure 6, D and E). The number of effector-like transitory CD8^+^ T cells in the mice receiving both treatments was approximately 10-fold lower than that in the mice treated with anti–PD-L1 antibody monotherapy. Consistent with this impaired CD8^+^ T cell response by mTOR inhibition, viral reduction was no longer observed in mice treated with anti–PD-L1 antibody plus rapamycin (Figure 6F). These data demonstrate that mTOR is required for PD-1–targeted immunotherapy to efficiently generate effector-like transitory T cells during chronic viral infection.

### PD-1–targeted immunotherapy works after cessation of mTOR inhibition.

**Figure 7:**
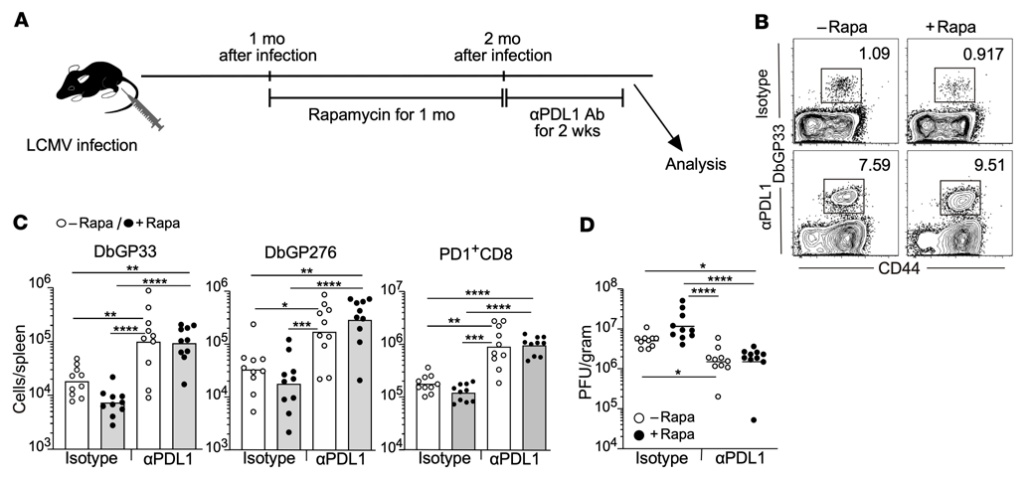
PD-1–targeted immunotherapy works after cessation of mTOR inhibition. (**A**) Experimental design. Mice were infected with LCMV clone 13, and then rapamycin treatment was started after establishing chronic infection. Rapamycin was administered daily i.p. to LCMV chronically infected mice from day 30 or 36 to day 61 or 66, respectively, for 1 month. PD-1 blockade was started 1 day after last rapamycin injection. Anti–PD-L1 antibody (αPDL1) or isotype control antibody (Isotype) was i.p. administered every 3 days, for a total of 5 injections (*n* = 10 per each group). Immune response and viral titer were analyzed 2 days after final injection of antibodies. (**B**) The frequency of DbGP33 tetramer^+^ CD8^+^ T cells in the spleen. Flow cytometry plots were gated on CD8^+^ T cells. (**C**) The number of DbGP33 tetramer^+^, DbGP276 tetramer^+^, and PD-1^+^CD8^+^ T cells in spleens. (**D**) Viral titer in the spleen. Each symbol represents an individual mouse. Each bar in **C** and each horizontal line in **D** represents geometric means. *P < 0.05; **P < 0.01; ***P < 0.001; ****P < 0.0001 (1-way ANOVA). Data were pooled from 2 independent experiments.

Although rapamycin treatment decreased the number of differentiated TIM3^+^CD8^+^ T cells in chronically infected mice, it minimally affected the quantity of stem-like CD8^+^ T cells (Figure 5). Because the stem-like CD8^+^ T cell population responds to PD-1–targeted immunotherapy (6), the efficacy of PD-1 blockade in rapamycin-treated mice may be comparable to that in control mice if mTOR inhibition does not alter the quality of stem-like T cells. To examine this, LCMV chronically infected mice were treated with rapamycin for 1 month, and then PD-1 blockade therapy was initiated after cessation of rapamycin treatment (Figure 7A). We found that the enhancement of antigen-specific CD8^+^ T cell numbers in rapamycin-treated mice after anti–PD-L1 antibody injection was indistinguishable from that in control mice (Figure 7, B and C). Thus, similar viral reduction was observed in both groups after PD-1 blockade (Figure 7D). These data indicate that rapamycin has minimal effect on the quantity and quality of stem-like CD8^+^ T cells, so that PD-1 blockade effectively activates antigen-specific CD8^+^ T cells after rapamycin treatment.

## Discussion

In this study, we found that mTOR inhibition has distinct effects on CD8^+^ T cell responses during the beginning of and after the establishment of chronic viral infection. Inhibition of the mTOR pathway during the clonal expansion phase promoted the formation of stem-like CD8^+^ T cells. Because stem-like CD8^+^ T cells can undergo self-renewal and continuously provide more-differentiated TIM3^+^ T cells (6), the accumulation of stem-like CD8^+^ T cells by rapamycin at the early phase of infection led to an enhanced quantity of antigen-specific CD8^+^ T cells as chronic infection progressed. Furthermore, consistent with the notion that stem-like CD8^+^ T cells respond to PD-1 blockade, the increase in the number of stem-like CD8^+^ T cells after mTOR inhibition improved efficacy of PD-1–targeted therapy. In contrast, when mice were treated with rapamycin in the late phase of chronic infection after fully establishing T cell exhaustion, the drug inhibited CD8^+^ T cell immunity by preventing stem-like T cells from differentiating into a TIM3^+^ state. This resulted in reduced numbers of TIM3^+^ more-differentiated CD8^+^ T cells accompanied by increased viral titer in rapamycin-treated mice. Thus, our findings demonstrate that mTOR plays a critical role in the progressive differentiation process of T cell exhaustion during chronic infection.

Several studies have attempted to examine the role of mTOR in T cell exhaustion by treating chronically infected mice with rapamycin (33, 34). However, it was unclear if rapamycin acted intrinsically in antigen-specific CD8^+^ T cells or whether the effect of rapamycin was due to another cell type because mTOR is ubiquitously expressed in many cells. For example, the response of antigen-specific CD4^+^ T cells that substantially influence development of CD8^+^ T cell exhaustion can be modulated by rapamycin (29). Here, our transcriptome analysis showed that rapamycin injection downregulated mTOR signaling–associated genes in antigen-specific CD8^+^ T cells. Furthermore, RNAi experiments revealed that the effect of rapamycin was lost in FKBP12 knockdown, rapamycin-insensitive, antigen-specific CD8^+^ T cells. These findings clearly establish that mTOR acts intrinsically in antigen-specific CD8^+^ T cells to regulate T cell exhaustion.

One of the most striking observations in our study was transcriptional upregulation of a group of ribosomal protein mRNAs in antigen-specific CD8^+^ T cells in rapamycin-treated mice. As previously reported (6, 7, 9, 19), we confirmed that, in the absence of rapamycin, stem-like CD8^+^ T cells expressed higher levels of ribosomal protein mRNAs compared with TIM3^+^ differentiated CD8^+^ T cells. Rapamycin treatment substantially enhanced expression of these transcripts in both subsets so that ribosomal protein mRNA levels in stem-like CD8^+^ T cells in the presence of rapamycin were highest among antigen-specific CD8^+^ T cell subsets that we examined. A similar transcriptional and translational change in expression of a large group of ribosomal protein transcripts was seen in antigen-specific CD8^+^ T cells during acute viral infection (43, 44). It will be interesting to examine how upregulation of ribosomal protein mRNAs by rapamycin affects the formation of ribosomes and whether translational regulation of gene expression is modulated in antigen-specific CD8^+^ T cells obtained from rapamycin-treated mice.

Our study has implications for PD-1–directed cancer immunotherapy. After the initial FDA approval of PD-1–blocking antibodies for the treatment of melanoma, the use of these antibodies has been extended to a wide variety of cancer types (2, 45). However, not all patients have a clinical response to this immunotherapy, and thus, there are considerable efforts focused on improving treatment outcomes. One promising approach is to combine the PD-1 blockade with other cancer therapies. Indeed, several PD-1 blockade combination therapies have shown better clinical efficacy and have been approved (45). A question now is whether mTOR inhibitors as well as the other anticancer drugs that inhibit molecules upstream of mTOR such as PI3K inhibitors can be used concurrently with PD-1 blockade to improve clinical response. PD-1 blockade induces proliferation of stem-like CD8^+^ T cells and, at the same time, promotes differentiation of stem-like CD8^+^ T cells into a TIM3^+^ state (6). During this differentiation process, proliferating antigen-specific CD8^+^ T cells acquire properties of effector cells, known as transitory T cells, to eliminate tumor cells (11, 19, 20). Although a previous work examined the effect of rapamycin during PD-1 blockade in chronically infected mice (34), it was not clear how mTOR inhibition affected individual antigen-specific CD8^+^ T cell subsets during PD-1–targeted therapy. We found that mTOR inhibition prevented PD-1 blockade–mediated induction of effector-like transitory CD8^+^ T cells from stem-like populations during simultaneous treatment, resulting in reduced efficacy of PD-1–targeted immunotherapy. These data suggest avoiding the concurrent use of mTOR inhibitors and PD-1 blockade in patients with cancer.

These findings raised the question of how mTOR is harnessed to improve PD-1–targeted immunotherapy. There are two potential approaches: (a) sequential administration of mTOR inhibitors and PD-1 blockade, and (b) mTOR activation only in antigen-specific T cells during PD-1 blockade. The first approach is based on our observations that, in contrast to TIM3^+^ differentiated CD8^+^ T cells, the quantity of stem-like CD8^+^ T cells was minimally affected by mTOR inhibition after establishing chronic infection. Therefore, sequential PD-1 blockade therapy after ceasing rapamycin treatment worked effectively, with an increase in antigen-specific CD8^+^ T cells and a decrease in viral loads. The same approach may maximize the effect of PD-1 blockade and mTOR inhibitors for cancer treatments. The idea of the second approach arose from our results, which illustrated that positive mTOR signals play an essential role in PD-1–targeted therapy. Thus, if drugs or procedures to increase mTOR activity in a T cell–specific manner are developed in the future, such novel treatments and PD-1 blockade may be used simultaneously to further enhance therapeutic efficacy in patients with cancer.

Our data also add insight regarding the use of adoptive cell immunotherapy (ACT), such as chimeric antigen receptor T cells (46, 47). Because a large number of these cells are required for successful treatment, they are stimulated and cultured in vitro to yield adequate quantity for ACT. Such stimulation can promote generation of cytotoxic effector cells accompanied by terminal differentiation. However, there is increasing evidence that less-differentiated T cells have better antitumor immune responses after adoptive transfer compared with terminally differentiated T cells (48). A possible explanation is that less-differentiated T cells are superior in terms of longevity and proliferative capacity compared with terminally differentiated T cells, thereby they survey for tumor cells longer and continuously provide cytotoxic effector cells once tumor cells are encountered. Considering our data in which rapamycin prevented terminal differentiation of T cells and induced accumulation of less-differentiated stem-like CD8^+^ T cells, the use of mTOR inhibitors during stimulation of T cells for ACT might yield a larger number of less-differentiated T cells.

The inhibitory effect of rapamycin on the generation of effector-like transitory CD8^+^ T cells from stem-like T cells implies a potentially new mechanism of mTOR inhibitor–mediated immunosuppression. Because continuous antigen stimulation promotes T cell exhaustion, organ transplantation could induce exhausted T cells. Indeed, several studies have suggested T cell exhaustion in response to allografts after transplantation (49–52). In addition, patients with cancer who were also transplant recipients rapidly developed allograft rejection after initiation of PD-1–targeted therapies (49), indicating the existence of allograft-specific exhausted CD8^+^ T cells that responded to PD-1 blockade in transplant recipients. In the transplant setting, T cell exhaustion could help to minimize T cell–mediated allograft rejection. Of note, mTOR inhibitors are used in organ transplant recipients to inhibit immune response against transplanted allografts. Although several potential mechanisms for mTOR inhibitor–mediated immunosuppression have been proposed, our results provide additional potential immunosuppressive mechanisms of mTOR inhibitors. Thus, rapamycin might exert an immunosuppressive effect by blocking the differentiation of antigen-specific CD8^+^ T cells from stem-like to effector-like transitory T cells.

Despite the clinical use of mTOR inhibitors, the role of mTOR in T cell exhaustion has not been well understood. Our results provide evidence that mTOR acts intrinsically in antigen-specific CD8^+^ T cells to regulate the progressive differentiation process of T cell exhaustion during chronic viral infection. The information obtained from our data can be applied to not only chronic infection but also cancer. Thus, our study has implications for the development of more effective treatment strategies for patients with cancer through targeting the mTOR pathway in antigen-specific CD8^+^ T cells.

## Methods

### Mice, viral infection, and viral titration.

Six- to 8-week-old female C57BL/6J mice were purchased from The Jackson Laboratory. P14 mice bearing the DbGP33-specific TCR were maintained in our animal colony. C57BL/6J mice were infected with 2 × 10^6^ PFU of LCMV clone 13 i.v. These LCMV-infected mice received 300 μg anti-CD4 antibodies (GK1.5; BioXCell, Leinco Technologies Inc. and in-house) i.p. on days 0 and 1 after infection for CD4^+^ T cell depletion in all experiments (53). Viral titers in the spleen and liver were measured by the plaque assay as described previously (54, 55).

### Administration of rapamycin.

Rapamycin (Pfizer, Apotex Corp., or Amneal Pharmaceuticals) was administered daily i.p. (600 μg per kg). In Supplemental Figure 3, 75 μg per kg of rapamycin was administered i.p. Blood concentration with these doses has been described previously (31). 600 μg per kg of rapamycin was used in most experiments, since this dose, although not statistically significant, showed a slightly better CD8^+^ T cell response compared with 75 μg per kg of rapamycin. Control mice received vehicle.

### PD-1–targeted therapy.

For PD-1 blockade therapy, 200 μg rat anti-mouse PD-L1 antibody (10F:9G2) or rat IgG2b isotype control (clone LTF-2 from BioXCell, clone 1–2 from Leinco Technologies Inc.) were administrated i.p. every 3 days, for a total of 5 injections.

### Cell sorting and RNA-Seq.

Cell sorting was performed with a FACSAria II or FACSAria Fusion (BD Biosciences). Single-cell suspensions were prepared from spleens of chronically infected mice treated with rapamycin or vehicle for 10 days from a day before infection, and TIM3^–^CXCR5^+^PD1^+^CD8^+^ T cells and TIM3^+^CXCR5^–^PD-1^+^CD8^+^ T cells were sorted (Supplemental Figure 4). Naive CD44^lo^CD8^+^ T cells were sorted from uninfected mice as a control. RNA was isolated from these sorted cells using Trizol reagent (Life Technologies) and RNA Clean and Concentrator-5 (Zymo Research). RNA-Seq was performed by Novogene Co. RNA-Seq data are available at the GEO database (accession GSE215248). RNA-Seq analysis was performed using Galaxy (56) or R. Mapping was performed with HISAT2 (Galaxy version 2.2.1^+^galaxy0) to align the reads with the mm10 genome (57), and read counts were generated with featureCounts (Galaxy version 2.0.1^+^galaxy1) (58). Differential gene expression analysis was performed using DESeq2 (59). PCA was performed by prcomp function with log-transformed data of normalized counts from DESeq2. The results were visualized by GraphPad Prism (version 9.2). Spearman’s correlation was calculated using normalized counts from DESeq2 and plotted by GraphPad Prism. For gene expression analysis in Figure 2C, we selected the top 3,000 differentially expressed genes among 4 populations (terminally differentiated TIM3^+^CXCR5^–^CD8^+^ T cells [rapamycin treated vs. control] and stem-like TIM3^–^CXCR5^+^CD8^+^ T cells [rapamycin treated vs. control]) (Supplemental Table 2). These genes were divided into 6 groups by k-means clustering (Numeric Clustering, Galaxy version 1.0.8.3) (60). The data were visualized in a heatmap using *z* scores. Genes in each cluster in Figure 2C were analyzed by Metascape (39) to determine overrepresented gene ontology categories. GSEA was performed using GSEA software (61, 62). Gene sets for ribosomes and cell cycle were obtained from the KEGG database (63–65), and a gene set for mTOR signaling was obtained from the Molecular Signatures Database Hallmark Gene Sets (66). Data from each cell population were compared with the combined data of the remaining 3 other populations.

### Retrovirus-based FKBP12 knockdown.

The knockdown of FKBP12 in antigen-specific CD8^+^ T cells (P14 cells) was performed using the pMKO.K.PGK.Thy1.1, in which SV40 promoter and GFP of pMKO.1.GFP (provided by W. Hahn, Harvard Medical School; Addgene plasmid 1067) were replaced with mouse PGK promoter and Thy-1.1, respectively. shRNA sequences targeting FKBP12 or scrambled shRNA were cloned into the retrovirus vector as described previously (31). For retrovirus transduction, P14 cells were activated in vivo by i.v. injecting 200 μg or 50 μg GP33 peptide into a P14 transgenic mouse treated with rapamycin. Splenic P14 CD8^+^ T cells were isolated and purified using the CD8^+^ T cell isolation kit (Miltenyi Biotech) 18–22 hours after peptide injection. Activated P14 cells were spin transduced with retrovirus as describe previously (31, 67). Transduced P14 cells were transferred into naive mice and rested in vivo for 3 days in the presence of rapamycin. Three days after in vivo resting, Thy1.1^+^ transduced P14 cells were sorted by a FACSAria II, FACSAria Fusion, SH800S, or MA900 (Sony Biotechnology). As a control, nontransduced P14 cells were prepared using the same method without retrovirus. Thy1.1^+^ transduced P14 cells and nontransduced P14 cells were mixed at a 1:1 ratio and cotransferred into naive mice, followed by infection with LCMV clone 13 and CD4 depletion with GK1.5 antibody.

### Proliferation and differentiation of virus-specific CD8^+^ T cells after establishing chronic infection.

To transfer stem-like CD8^+^ T cells into LCMV-infected mice after establishing chronic infection, TIM3 CXCR5^+^PD1^+^CD8^+^ T cells were sorted from spleens of chronically infected mice (CD45.2^+^) on day 42 or 43 after infection. 4 × 10^4^ to 5 × 10^4^ sorted cells were adoptively transferred into chronically infected recipient mice (CD45.1^+^) on day 31 or 33 after infection. The water for these recipient mice was supplemented with 0.8 mg/mL BrdU, and mice were treated or not with rapamycin every day from 1 day before stem-like CD8^+^ T cell transfer. BrdU incorporation into the transferred CD8^+^ T cells (CD45.2^+^) was measured by a BrdU flow kit (BD Biosciences). To detect CD45.2^+^ transferred cells in the recipients on day 10 after adoptive transfer, CD45.2^+^ splenocytes were enriched by anti–CD45.2-APC combined with anti-APC MicroBeads (Miltenyi Biotech).

### Adoptive transfer of stem-like CD8^+^ T cells generated in the presence or absence of rapamycin.

To transfer stem-like CD8^+^ T cells, TIM3^–^CXCR5^+^PD1^+^CD8^+^ T cells were sorted from LCMV chronically infected mice (CD45.2^+^) treated with rapamycin or vehicle. 25 × 10^3^ to 50 × 10^3^ cells of sorted cells were transferred into infection-matched recipient mice (CD45.1^+^). Anti–PD-L1 antibody i.p. injection was started 1 day after transferring stem-like CD8^+^ T cells (every 3 days, for a total of 5 injections). At day 14 after PD-1 blockade, CD45.2^+^ cells in spleens and livers were enriched using anti–CD45.2-APC combined with anti-APC beads (Miltenyi Biotech) for analysis.

### In vitro culture of P14 CD8^+^ T cells.

To obtain P14 CD8^+^ T cells derived from LCMV chronically infected mice, P14 CD8^+^ T cells from spleens of P14 TCR transgenic mice were adoptively transferred into C57BL/6J (B6) mice (2 × 10^3^ P14 cells per mouse). These mice were i.v. infected with 2 × 10^6^ PFU of LCMV clone 13 1 day after adoptive transfer of P14 cells, and they also received 300 μg anti-CD4 antibodies (GK1.5) i.p. on days 0 and 1 after infection. Splenic single-cell suspensions were prepared from these mice after establishing T cell exhaustion (>30 days after infection). To obtain splenic single-cell suspensions containing naive P14 CD8^+^ T cells, P14 CD8^+^ T cells were adoptively transferred into B6 mice (2 × 10^6^ P14 cells per mouse), and spleens were isolated 1 day after transfer. Both spleens obtained from LCMV chronically infected and uninfected mice contained P14 cells at similar frequencies. Using 24-well plates, splenic single-cell suspension (3 × 10^6^ cells per well) was stimulated with the stimulation RPMI1640 medium (Gibco) in the presence or absence of rapamycin (Sigma-Aldrich) for 7 days. The stimulation RPMI1640 medium contained GP33 peptide (1 ng/mL), IL-2 (50 U/mL, R&D Systems), fetal bovine serum (10%), sodium pyruvate (1 mM, Gibco), nonessential amino acids (100 μM, Gibco), Antibiotic-Antimycotic (1×, Gibco), 2-mercaptoethanol (50 μM, MP Biomedicals), and L-glutamine (2 mM). A 4-fold serial dilution of rapamycin was added into the wells at the time of stimulation.

### Flow cytometry.

Cells were isolated in the spleen, liver, lung, and peripheral blood as described previously (54). To stain cell surface, cells were incubated with an antibody cocktail for 30 minutes on ice and then fixed by the Fixation/Permeabilization Solution Kit (BD Biosciences). For intracellular staining of TCF1, the Foxp3/transcription factor staining buffer set (eBioscience) was used. LCMV MHC class I tetramers were made and used as previously described (54, 68). The following antibodies were purchased from BioLegend: CD44 (clone IM7), CD45.1 (clone A20), CD8 (clone 53-6.7), CXCR5 (clone L138D7), PD-1 (clone 29F.1A12 or RMP1-30), TIM3 (clone RMT3-23), CX3CR1 (clone SA011F11), CD45.2 (clone 104). The following antibodies were purchased from BD Biosciences: CD8 (clone 53-6.7) and TCF1 (clone S33-966). PD1 (clone RMP1-30) antibody was purchased from eBioscience. TIM3 (clone 215008) antibody was purchased from R&D Systems. TCF1 antibody (clone C63D9) was purchased from Cell Signaling. Live/Dead near IR (Invitrogen) was used to gate out dead cells. LSRII and CANTO (BD Biosciences) were used to acquired flow cytometry data, which were analyzed by Flowjo version 10 (BD Biosciences).

### Statistics.

*P* values were calculated by 2-tailed unpaired *t* test for 2 groups and 1-way ANOVA for 3 or 4 groups using log-transformed values except for statistical analyses of percentages. *P* values for percentages were determined by 2-tailed paired *t* test for the FKBP12 knockdown experiment and by 2-tailed unpaired *t* test for other experiments. Statistical analysis was performed on GraphPad Prism 9 software. *P* values of less than 0.05 were considered significant.

### Study approval.

All mice were used in accordance with animal protocols approved by Institutional Animal Care and Use Committee at Emory University and Cincinnati Children’s Hospital Medical Center.

## Author contributions

SA, MH, SSR, RA, and KA designed experiments. SA, CMP, YS, CC, RMV, AW, and KA performed experiments. SA, WHH, MH, SSR, RA, and KA analyzed data. GJF contributed critical materials. SA, CMP, and KA wrote the manuscript.

## Supplementary Material

Supplemental data

Supplemental table 1

Supplemental table 2

Supplemental Conflict of interest statement

## Version 1 11/15/2022

In-Press Preview

## Version 2 01/17/2023

Electronic publication
