# Supplementary material for: mTOR regulates T cell exhaustion and PD-1–targeted immunotherapy response during chronic viral infection
Source: J Clin Invest. 2023 Jan 17;133(2):e160025. doi: 10.1172/JCI160025 (PMC9843061; doi:10.1172/JCI160025)
Supplement: Supplemental data [file jci-133-160025-s043.pdf]

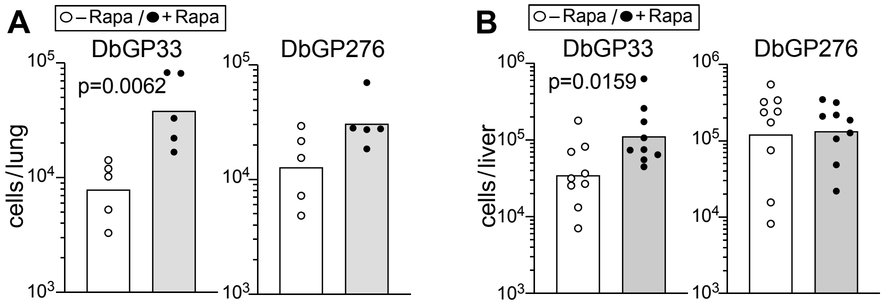

Supplemental Figure 1. LCMV-specific CD8 T cells in lung and liver. Mice were infected with LCMV clone13 in the presence or absence of rapamycin treatment. Rapamycin was injected intraperitoneally every day from day -1 to days 30-35 post infection and then LCMV-specific CD8 T cell response was examined on days 31-36 after infection. The number of DbGP33 tetramer$^+$ and DbGP276 tetramer$^+$ CD8 T cells in lung (A, n=5 per each group) and liver (B, n=9 per each group). Each symbol represents an individual mouse. Each bar represents geometric means and p-values were calculated by unpaired t-test. Data were pooled from two or three independent experiments.

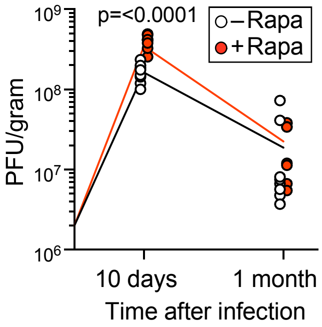

Supplemental Figure 2. Viral titers in spleen obtained from rapamycin-treated LCMV-infected mice. Mice were infected with LCMV clone13 in the presence or absence of rapamycin treatment. Rapamycin was injected intraperitoneally every day from day -1 to day 30-39 (1 month) after infection. Viral titers were measure in spleen on days 10 and 31-36 (1 month) post infection. n=8 for each group on each time point. Each symbol represents an individual mouse. Each line represents geometric means and p-values were calculated by unpaired t-test. Data were pooled from two or three independent experiments.

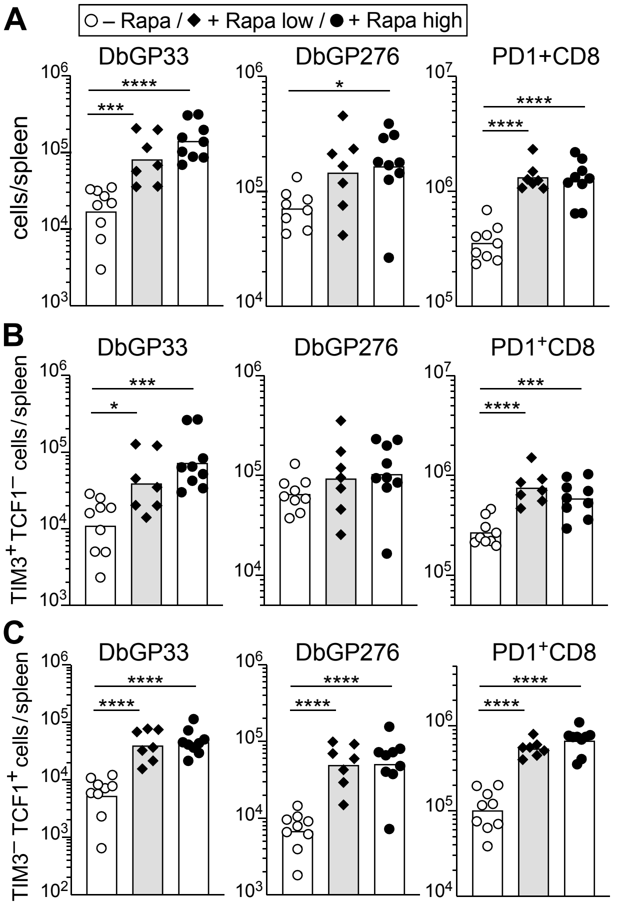

Supplemental Figure 3. Low dose of rapamycin enhances CD8 T cell responses during the early phase of chronic infection. Mice were infected with LCMV clone13 in the presence of low dose of rapamycin (75 µg per kg, closed diamonds). Rapamycin was injected intraperitoneally every day from day -1 to days 29-34 post infection and then LCMV-specific CD8 T cells response was examined on day 30-35 after infection. (A) The number of total DbGP33 tetramer$^+$, DbGP276 tetramer$^+$ and PD1$^+$ CD8 T cells in spleen (B) The number of TIM3$^+$, differentiated (TIM3$^+$ TCF1$^-$) or (C) stem-like (TIM3$^-$ TCF1$^+$) CD8 T cells in spleen is shown for DbGP33 tetramer$^+$, DbGP276 tetramer$^+$, and PD-1$^+$ CD8 T cells (n=7 for rapamycin low dose group). For a comparison, Figure 1 data obtained from untreated (open circles) and high-dose rapamycin-treated mice (600 µg per kg, closed circles) are shown. Each symbol represents an individual mouse. Each bar represents geometric means. *, $p < 0.05$; **, $p < 0.01$; ***, $p < 0.001$; ****, $p < 0.0001$ (one-way ANOVA). Data was pooled from two or three independent experiments.

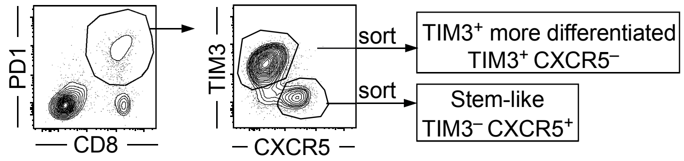

Supplemental Figure 4. Cell sorting strategy for Figure 2. LCMV clone 13-infected mice were treated or not with rapamycin from day -1 to day 9. At day 10 post infection, stem-like (TIM3^{-} CXCR5^{+} PD-1^{+}) or TIM3^{+} differentiated (TIM3^{+} CXCR5^{-} PD-1^{+}) CD8 T cells were sorted, and RNA-seq was performed.

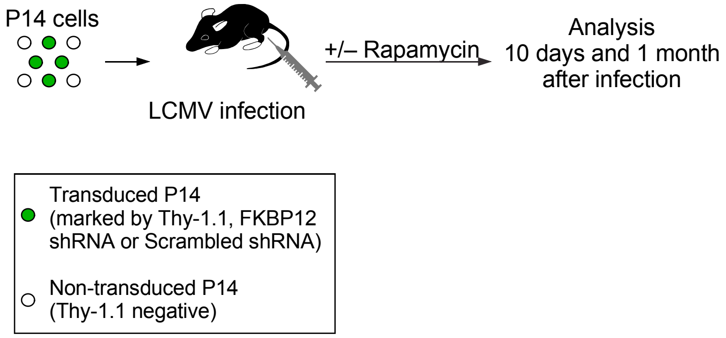

Supplemental Figure 5. Experimental design for Figure 3 and Supplemental Figure 6. LCMV-specific transgenic CD8 T cells (P14 cells) were transduced with retrovirus expressing shRNA for FKBP12 or scrambled shRNA. These retrovirus-transduced P14 cells (marked by Thy1.1) and non-transduced P14 cells were adoptively co-transferred into rapamycin treated B6 mice, followed by LCMV clone13 infection. Rapamycin was intraperitoneally administered every day from day -1 to 30 post infection.

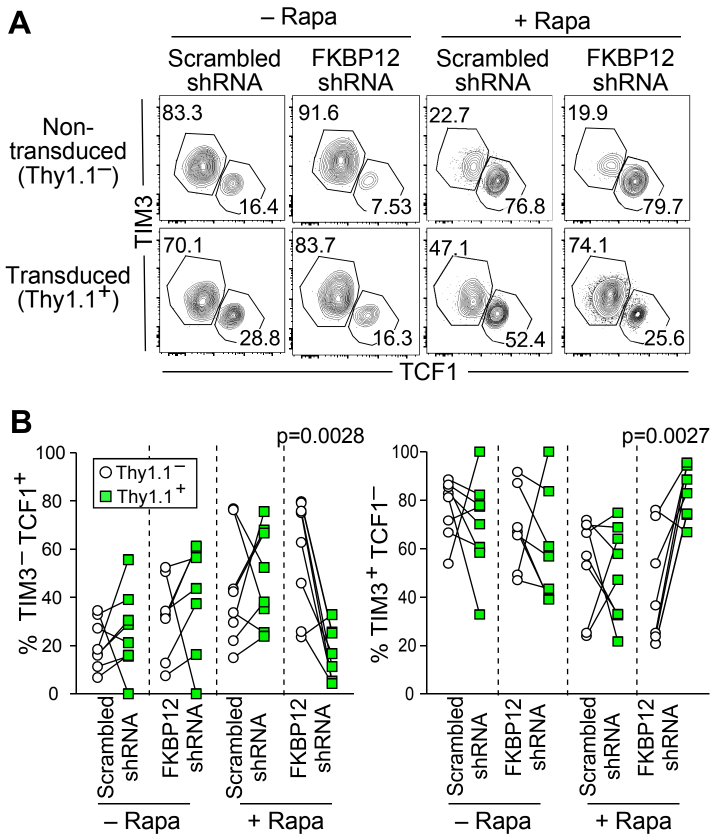

Supplemental Figure 6. Phenotype of P14 cells transduced with retrovirus expressing FKBP12 shRNA or scrambled shRNA at 1 month post infection. Experiments were performed as described in Supplemental Figure 5. P14 cells were analyzed at day 30 or 31 post infection. (A) The flow cytometry plots, gated on retrovirus-transduced (Thy1.1$^+$) or non-transduced (Thy1.1$^-$), show the frequency of stem-like (TIM3$^-$ TCF1$^+$) and TIM3$^+$, differentiated (TIM3$^+$ TCF1$^-$) retrovirus-transduced and non-transduced P14 T cells in spleen. (B) The frequency of stem-like (TIM3$^-$ TCF1$^+$, left) and TIM3$^+$, differentiated (TIM3$^+$ TCF1$^-$, right) CD8 T cells in retrovirus- transduced (green square) and non-transduced (white circle) P14 T cells in spleen. n=8 per each group except for the following group: FKBP12 shRNA without rapamycin (n=7). Each line represents a comparison between non-transduced and transduced P14 cells in the same mice. Data were pooled from three independent experiments. P values were calculated by paired t-test.

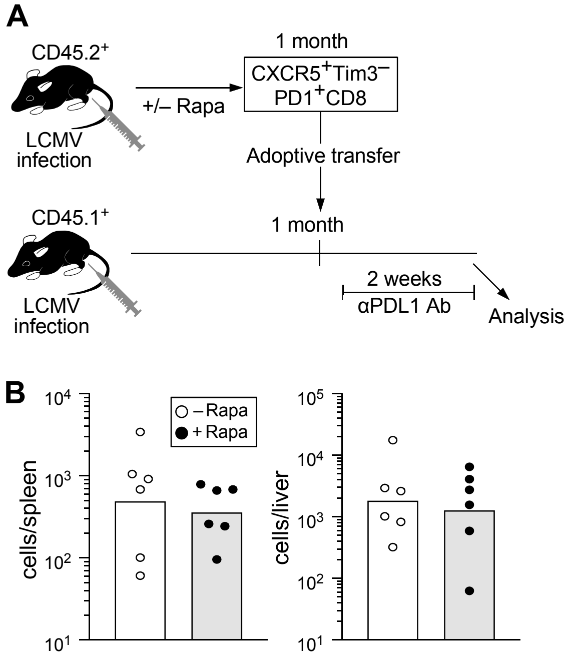

Supplemental Figure 7. The responsiveness of stem-like CD8 T cells generated in the presence or absence of rapamycin to anti-PD-L1 antibody treatment. (A) Experimental design. Mice ($CD45.2^{+}$) were infected with LCMV Clone13 in the presence or absence of rapamycin. Rapamycin was injected intraperitoneally every day from day -1 of infection and then stem-like CD8 T cells ($TIM3^{-}$ $CXCR5^{+}$ $PD1^{+}$) in spleens obtained from rapamycin treated or untreated mice were sorted around 1 month post infection (day 30 or 31). Sorted cells were adoptively transferred into infection-matched chronically infected mice (day 30 or day 31 after infection, $CD45.1^{+}$). Anti-PD-L1 antibody (aPDL1) treatment was started the day after adoptive transfer, and the antibody was intraperitoneally administered every 3 days, 5 total injections (n=6 per each group). (B) Total number of transferred LCMV-specific CD8 T cells ($PD1^{+}$ $CD45.2^{+}$) in spleen (left) and liver (right). Each symbol represents individual mouse (white circle; stem-like CD8 T cells generated in the absence of rapamycin, black circle; stem-like CD8 T cells generated in the presence of rapamycin). Each bar shows geometric means. Data were pooled from three independent experiments.

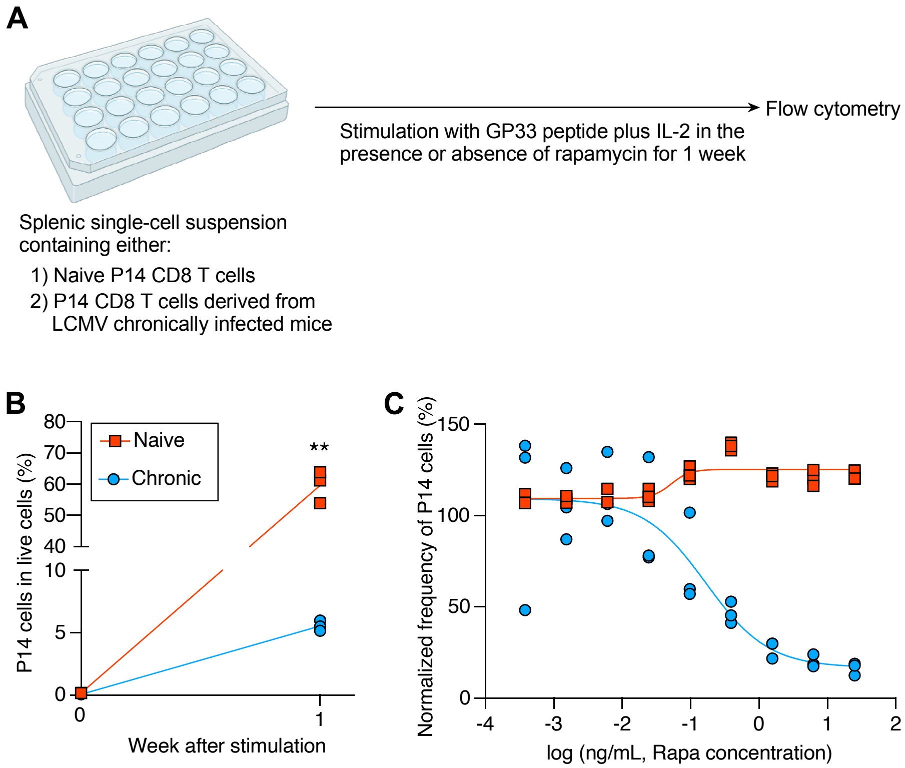

Supplemental Figure 8. Proliferative capacity and susceptibility to mTOR inhibition differ between naïve and chronically stimulated CD8 T cells. (A) Experimental design. Splenic single-cell suspension containing LCMV-specific transgenic CD8 T cells (P14 cells) was prepared. P14 cells were derived from either uninfected P14 transgenic mice (Naïve) or LCMV chronically infected mice (Chronic) in which naïve P14 cells were adoptively transferred prior to infection. Individual culture plate wells of the splenic single cell suspension contained equal numbers of P14 cells. Cells were stimulated with GP33 peptide plus IL-2 in the presence or absence of rapamycin for 1 week and the expansion of P14 cells were examined by flow cytometry. Created with BioRender.com. (B) Expansion of P14 cells 1 week after stimulation without rapamycin. (C) The effect of rapamycin on the expansion of P14 cells is shown. The average frequency of expanded P14 cells 1 week after stimulation without rapamycin was set as 100% for normalization. Individual symbols in B and C indicate three technical replicates and lines in B indicate means. Non-linear regression lines for Naïve and Chronic are shown in C. P-value was calculated by unpaired t-test. Data are representative of two independent experiments.
